# Fish as Food

**Published:** 1889-12-28

**Authors:** 


### DIETETICS.

### FISH AS FOOD.

A contemporary recently observed that sometimes attacks of diarrhœa follow the ingestion of oysters, but the comparative rarity of this leads many to brave the danger with an easy mind. Bowel irritation following eating oysters is more frequently observed in tropical and semi-tropical than in temperate climates. In Bombay, for instance, such affections following oysters as a food are so frequent that many Anglo-Indians will not use them. Recently, in Dublin, a correspondence has taken place in the local newspapers as to the probability of typhoid fever being occasioned by eating oysters, due to the presence of sewage matter in the oyster or its juices. This is quite possible, although we think not probable. In Bombay some time ago it was theorised that oysters taken from mangrove swamps were more deleterious than those obtained from places where the mangrove did not grow. This, however, was proved to be fallacious, and the idea that mangrove swamps collected or impeded the flow of sewage became untenable, and the unhealthiness of oysters was perforce attributed to commencing decomposition. It is not, however, oysters only which produce diarrhœa or other unpleasant results. An inquest was recently held in London by Dr. Thomas, where illness and death appear to have resulted from partaking of fried plaice. Shell-fish are often credited with exciting diarrhœa, indigestion, nettle-rash, and other ailments. Occasionally, especially in tropical climates, the symptoms produced by impure fish are very much the same as those of cholera. A fish which may be perfectly fresh and wholesome at one time of the day may, on peculiar conditions of the atmosphere, be the reverse in a few hours. Doubtless this depends on the formation of a ptomaine possessing poisonous properties. Such a poisonous principle has, in fact, been isolated from shell-fish by Brieger, who terms it mytilotoxine. Inferior and cheap fish are constantly being hawked about the streets, and perhaps even sold in shops. Often a fish with ptomaine inside may be sold in ignorance of any such change having taken place. Mr. J. Lawrence Hamilton tells us that occasionally stale cod fish have their gills smeared with fresh bullock's blood, so that they may appear recently captured. Fish are sometimes inflated with air or "blown," so as to make skinny fish appear plump and fat. Small haddocks and rock pouters are often skinned, and their tails inserted in their mouths, and sold at a high price for whiting. Filleted soles are often dressed weavers or plaice. Halibut and brill are passed off as turbot. Turtle soup is often manufactured from conger eel. Lobsters are freshened up by reboiling, thus concealing the odour of commencing decomposition. Light weight lobsters are made heavy by the introduction of pieces of fresh haddock. Canned oysters are often mussels with their tyssus or moorings removed: and so on throughout the whole of the fish trade, deception seems to be practised in order to dispose of this perishable article of food. We certainly had little idea of the extent of the deception practised, and cannot but hope Mr. J. Lawrence Hamilton, in his "Fish Frauds," describes only exceptional occurrences. The fish thus deteriorated are chiefly sold to the poor, whose fresh food supplies are always dear, and too frequently unwholesome and unpalatable, acting as one of the overlooked factors which drive many to chronic intemperance. We think, however, that something must be attributed to the treatment the fish receives after it is bought by the poor. Those acquainted with the domiciles of the poor will readily understand that a fish kept only a few hours in certain places would be in the most favourable position possible for the formation of ptomaines, often the prelude of commencing decomposition. Then there is bad cooking, many, perhaps most, of the wives of working-men having too little knowledge of this essential point of household economy. Fish is a most important article of food supply, and under a better system good fish might be rendered much more available to the poor than it now is. We have heard of quantities of fish being thrown back into the sea in order to maintain the price. Something should certainly be done to prevent any such waste. And it would also seem that more stringent regulations are required to prevent the sale of fish more or less unfit for human food. At present, there are no certain means of ascertaining if a fish has developed a ptomaine or not, for decomposition sufficient to be recognised by the smell is not essential. It is possible, or even probable, that ere long this object may be accomplished, and that fish of a deleterious nature will not then be either knowingly or ignorantly sold for food.

